# The role of law and governance reform in the global response to non-communicable diseases

**DOI:** 10.1186/1744-8603-10-44

**Published:** 2014-06-05

**Authors:** Roger S Magnusson, David Patterson

**Affiliations:** 1 Sydney Law School, F10, The University of Sydney, Sydney NSW 2006 Australia; 2 Department of Strategy and Innovation, International Development Law Organization (IDLO), Viale Vaticano, Rome, Italy

## Abstract

Addressing non-communicable diseases (“NCDs”) and their risk-factors is one of the most powerful ways of improving longevity and healthy life expectancy for the foreseeable future – especially in low- and middle-income countries. This paper reviews the role of law and governance reform in that process. We highlight the need for a comprehensive approach that is grounded in the right to health and addresses three aspects: preventing NCDs and their risk factors, improving access to NCD treatments, and addressing the social impacts of illness. We highlight some of the major impediments to the passage and implementation of laws for the prevention and control of NCDs, and identify important practical steps that governments can take as they consider legal and governance reforms at country level.

We review the emerging global architecture for NCDs, and emphasise the need for governance structures to harness the energy of civil society organisations and to create a global movement that influences the policy agenda at the country level. We also argue that the global monitoring framework would be more effective if it included key legal and policy indicators. The paper identifies priorities for technical legal assistance in implementing the *WHO Global Action Plan for the Prevention and Control of NCDs 2013–2020*. These include high-quality legal resources to assist countries to evaluate reform options, investment in legal capacity building, and global leadership to respond to the likely increase in requests by countries for technical legal assistance. We urge development agencies and other funders to recognise the need for development assistance in these areas. Throughout the paper, we point to global experience in dealing with HIV and draw out some relevant lessons for NCDs.

## Background

Nearly two out of every three deaths globally are caused by chronic, non-communicable diseases (“NCDs”), including cardiovascular disease, cancer, diabetes and chronic lung diseases [1-3]. The modifiable causes of the leading NCDs are well established: tobacco use, harmful use of alcohol, obesity, lack of physical activity, and excess salt, sugar and saturated fats in the diet [4-6]. About one quarter of global NCD deaths occur before the age of 60, undermining productivity and economic growth, especially in low- and middle-income countries – where 80% of all deaths occur, where deaths occur at a younger age, where safety nets are fragile or absent, and where treatment costs are prohibitive [4]. A study conducted for the World Economic Forum estimates that under a “business as usual” scenario, low- and middle-income countries could lose $500 billion per year over the period 2011–2025 due to NCD morbidity and mortality: roughly 4% of average GDP for these countries [7]. Addressing NCDs and their risk factors is not merely a priority for economic development: it is likely to be the most powerful way of improving longevity and healthy life expectancy for the foreseeable future – especially in low- and middle-income countries.

Recognising these factors, in September 2011 the United Nations General Assembly held a high-level meeting on NCDs (“UNGA High-level Meeting”) [8]. In the lead-up to this meeting, there was a significant level of agreement about the low cost, cost-effective, feasible and evidence-informed interventions that could most effectively reduce the burden of NCDs in low- and middle-income countries [4,5,7,9,10]. Summarised in Table 1, these priority interventions or “best buys” focus principally on tobacco cessation, moderating alcohol intake, improving diet, and encouraging higher levels of physical activity. These priorities have been reiterated in the *WHO Action Plan for the Prevention and Control of NCDs 2013–2020*[11] and elsewhere in the literature [12,13]. Together, these measures build on the *WHO Framework Convention on Tobacco Control* (“FCTC”) [14], the *WHO Global Strategy to Reduce the Harmful Use of Alcohol*[15], the *WHO Global Strategy on Diet, Physical Activity and Health*[16], and other strategies [17,18]. Without substantial progress in implementing these measures, countries are unlikely to meet the global target of a 25% reduction in NCD mortality by 2025 (“25 by 25”), adopted at the World Health Assembly in May 2012 [19].

**Table 1:** **Prevention: legal and regulatory priorities for reducing major risk factors for non-communicable diseases within the population**[4]

Tobacco	Comprehensive implementation of the *WHO Framework Convention on Tobacco Control* (WHA56.1) especially:
	● Imposing and increasing excise taxes on tobacco to reduce demand (FCTC Article 6) **[BEST BUY]**;
	● Smoking bans in public places, including workplaces, public transport, bars and restaurants (FCTC Article 8) **[BEST BUY]**;
	● Health warnings on tobacco products, and at point of sale; labelling controls (FCTC Article 11,12) **[BEST BUY]**;
	● Comprehensive bans on tobacco advertising, promotion and sponsorship, including in all media, in community settings, and in retail establishments (FCTC Article 13) **[BEST BUY]**;
	● Bans on sales of tobacco to and by children, with monitoring and enforcement (FCTC Article 16);
	● Penalties for smuggled and counterfeit tobacco; with adequate resources for monitoring and enforcement (Article 15; protocol to eliminate illicit trade in tobacco products);
	● Affordable treatment for tobacco dependence: supporting interventions for smoking cessation in primary care; affordable pharmacological therapies (FCTC Article 14);
Alcohol	Implementation of the *WHO Global Alcohol Strategy* (A63/13), especially:
	● Increasing excise taxes on alcoholic beverages (paras. 32–34) **[BEST BUY]**;
	● Penalties for smuggled and informal alcohol, with adequate resources for monitoring and enforcement (paras. 37–39);
	● Restrictions on alcohol advertising and promotion through the media, in community settings and retail establishments; restrictions on alcohol sponsorship of cultural and sporting events (paras. 29–31) **[BEST BUY]**;
	● Controls on access to retailed alcohol, including minimum age purchasing laws, licensing and other controls on hours of retail sale, location and density of retail outlets (para. 27–28) **[BEST BUY]**;
	● Health warnings on alcohol products and at point of sale (paras. 19, 36);
	● Drink-driving counter-measures, including random breath testing, a maximum 0.5 g/l blood alcohol concentration (BAC) limit for adult drivers, with a reduced or zero limit for younger drivers (paras. 24–26);
	Building on the *WHO Global Strategy on Diet, Physical Activity and Health* (WHA57.17):
	● Institutional and governance reform to enable development of a comprehensive and multi-sectoral approach to policy development for diet, nutrition and physical activity, with input from key sectors (agriculture, transport, education, environmental and urban planning, sport, youth, industry, finance, and media and communications). City and local governments should have a legal mandate to play a leading role (paras. 38–44);
Diet, and physical activity	● Measures to reduce salt levels in food, such as encouraging food reformulation through public reporting of food manufacturers’ commitments to progressive reductions (para. 41); measures to replace saturated with unsaturated fats in food products;
	● Requiring food manufacturers to replace trans fats with polyunsaturated fats (para. 41) [4]**[BEST BUY]**;
	● Restrictions on marketing of foods and beverages high in salt, sugar and fats (especially to children): WHO, Set of recommendations on marketing of foods and non-alcoholic beverages to children WHA 64.14, adopted May 2010);
	● Improving food labelling to encourage healthier choices;
	● Fiscal measures such as reduced taxation on healthier foods, and/or higher taxation for foods to be consumed in lower quantities (para 41);
	● Legislation to protect women’s right to breast-feed, without harassment or discrimination [4].
Other strategies	● Hepatitis B vaccination.

A striking feature of these priority interventions is their reliance on legal and regulatory reforms. They are either fiscal policies (e.g. raising taxes), or their successful implementation will depend, in many cases, upon legislation or subsidiary statutory instruments (regulations) that prescribe standards, mandate required actions and processes to be followed, and require government agencies to play a central role in monitoring and enforcement. This does not mean, of course, legal and governance strategies are the only strategies for controlling NCDs. A comprehensive approach to encouraging healthier lifestyles will also include community-based programs, health promotion, and a greater focus on prevention within primary care. Investment in health care systems – including infrastructure and the health workforce – together with monitoring, reporting and accountability mechanisms, and political leadership at the highest level, are also critical elements for successful national approaches [5].

On the other hand, attempts by governments to “spend their way out” of the NCD crisis by investing in treatment services alone will be futile unless matched by policies that target the whole population, address the shared risk factors for the leading NCDs, and empower individuals and communities to lead healthier lives [9]. Prevention is vital to the control of NCDs, and law is an important tool for achieving this goal.

This paper reviews the role of law and governance reform in the implementation of successful national responses to NCDs, particularly in low- and middle-income countries. We highlight the need for governments, NGOs, development agencies and other stakeholders to recognise the importance of law in strategies for both prevention and treatment of NCDs, and to plan for greater investment in legal capacity-building in low- and middle-income countries. While there is considerable agreement about legislative priorities for prevention, law’s role in improving access to treatment for NCDs is more variable and country-specific, but relates closely to the challenge of strengthening primary health care systems. In addition, we highlight the role of law in addressing the social impact of illness, including its role in preventing discrimination against people with diabetes and other NCDs [20]. Given that the leading NCDs share a cluster of inter-related yet modifiable risk factors, there are benefits in adopting a national strategy that requires countries to set out their responses to all the major risk factors. By focusing on the leading NCDs and their risk factors generally, it is less likely that major risk factors will be ignored. At the same time, countries will still need to make choices about priorities [21], and it may be important to protect the work already undertaken at ational level in specific areas, such as tobacco control.

Action on all three fronts – prevention of key risk factors, treatment for NCDs, and addressing the social impacts of illness – makes sense in policy terms by reducing future expenditures, improving productivity, and reducing suffering. In addition, however, taking actions on these fronts is part of the obligation that many countries have undertaken to respect, protect and fulfil the right to health. First recognized in the Constitution of the World Health Organization [22], the right to health is enshrined in six core international human rights treaties including the International Covenant on Economic, Social and Cultural Rights (“ICESCR”) [23]. It is also recognised in three regional human rights agreements [24-26], and in national constitutions in some countries, such as Brazil [27].

Having identified priorities for NCD-related law reform, this paper points to some of the most difficult challenges that governments face when using legal strategies to prevent and manage NCDs. We identify key practical steps that governments can take as they consider law and governance reforms at country level. Finally, we consider global architecture for NCDs, accountability for law and governance reforms, and priorities for global leadership in NCD-related law and governance reform in future.

There is a great deal to be learned from 30 years of experience in responding to HIV in developing countries [28]. The leading NCDs have important similarities with HIV, including the fact that both are transmitted through human behaviours that are modifiable, yet largely determined by cultural, social and economic factors. HIV and NCDs both impact heavily on people during their productive years, both require an inter-sectoral response from government that addresses both prevention and treatment, and both depend on an enabling legal environment. As with HIV, the burden of caring is more likely to fall on women, who also become the sole breadwinner when a male partner falls ill. Without adequate national or private health insurance, treatment costs may rapidly deplete household savings, pushing families into poverty and forcing children, particularly girls, to quit school [29,30]. Throughout the paper, we draw attention to how the global governance of HIV might inform law reform and governance processes for NCDs.

## Law reform priorities for the control of NCDs: balancing prevention and treatment

Low- and middle-income countries looking to respond to the rising burden of cardiovascular disease, diabetes and tobacco-related diseases are confronted by many challenges. One of the most immediate is to balance investments in the delivery of healthcare services to those who are already sick with attention to the preventive policies that will largely benefit future generations. The scope and scale of public health law reform for NCDs in each country will reflect the balance that is struck between prevention, and treatment.

The case for prevention is based on the fact that the rising burden of NCDs in low- and middle-income countries is mostly attributable to shared risk factors that are modifiable through public policies that target the population generally, as well as high-risk individuals [9]. Although estimates vary, over 2.6 million of the 7.6 million annual cancer deaths [31] and, theoretically, all tobacco-related deaths, could be avoided through the implementation of preventive policies targeting known risk factors. The case for prevention also depends, implicitly, on the consequences of inaction. For example, the number of adults with diabetes is expected to rise from 285 million to 439 million between 2010 and 2030, with a 69% increase in developing countries [32]. Taking preventive action now is vital to the capacity of countries to afford the costs of treatment for their populations in future.

Affordable and cost-effective policies for reducing NCD risk factors are available and have been described, including salt reduction [5,7,33-35] and implementing countries’ obligations under the FCTC [14]. For example, Cecchini and colleagues estimated that a basket of mostly population-level preventive policies in six middle-income countries could be implemented at a cost of US$1.5-4.5 per person [35]. This is a cost of less than 1% of total per capita health spending within countries including China, Mexico, Brazil and Russia [9]. At US$11.4 billion per year, the total cost of a set of “best buy” interventions for prevention and treatment looks relatively modest in the sense that it amounts to an investment of less than US$1 per person per year in low-income countries, less than US$1.50 in lower middle-income countries, and US$3 in upper middle-income countries [33]. The overwhelming majority of these costs are made up of screening and drug treatment costs for high-risk individuals, rather than the less expensive, population-based, preventive interventions that focus on reducing tobacco use and harmful use of alcohol, improving diet, and encouraging physical activity [33].

Although it makes little sense for governments to invest almost exclusively in treating illness when affordable policies are available to reduce the scale of treatment costs in future, the tendency for prevention to languish in national budgets is well recognised. The costs of neglecting prevention can be deferred – at least temporarily. There may also be more immediate political pressures to respond to patients who are sick now, and these costs alone may outstrip limited budgets. An additional factor is that prevention cannot be achieved simply by spending more. Table 1 summarizes the World Health Organization (“WHO”)’s priority recommendations for NCD prevention, including “best buys”. In order to implement these recommendations, governments must deal with coordinated campaigns from industry lobby groups (tobacco, alcohol and food manufacturers and retailers), and risk challenges from trading partners appealing to World Trade Organization (“WTO”) agreements, regional trade agreements, and bilateral investment treaties (“BITs”) [36-38]. Population-wide preventive policies cannot easily be implemented without national political leadership at the highest level, since these policies relate to a range of non-health sectors and must be implemented by non-health Ministries. For these reasons, effective NCD prevention requires a multi-sectoral, “all-of-government” approach, ideally led by a President or Prime Minister [39-42]. The creation of new national structures requires considerable political effort and broad political support: this is why, in our view, accountability mechanisms for NCDs need to monitor actions taken at the national level to implement specific legal and policy measures, in addition to monitoring national trends in risk factors and disease outcomes.

NCDs raise similar challenges to the HIV epidemic: prevention calls for significant changes in the behaviour of populations, and in the case of some NCDs, requires the provision of lifelong access to essential medicines. An important lesson learned from HIV programs, however, was that a two-pronged approach that integrates treatment and prevention is superior to an approach that prioritises prevention alone [43]. There are several reasons for this. People living with HIV have long been powerful advocates for policy and law reform [44]. The lifestyle and behavioural changes that are required to prevent transmission benefit from peer education and personal testimony from persons infected. Drug treatment for HIV has also been shown to prevent new infections as well as delay disease progression [45]. In societies where the treatment needs of those with HIV – and NCDs – are acknowledged and resourced, prevention is more likely to be effective. In societies where there is no realistic prospect of treatment, there is no evidence to suggest that individuals are more likely to adopt the behaviours that will prevent them from falling sick. Another lesson from HIV is the importance of considering gender in policies aimed at prevention and treatment. For example, cultural restrictions may limit girls’ access to sporting facilities, or create invisible barriers to a physically active lifestyle. Further, in some cultures, women and girls’ diets are poorer than their male counterparts, even within the same household [46-48].

An effective response to NCDs requires governments to adopt cost-effective and mutually reinforcing measures for both prevention and treatment [4,5,10], while also addressing discrimination and the social impacts of illness. Treatment *is* prevention in the case of life-long, chronic conditions and risk-factors that – like obesity, hypertension, high cholesterol and diabetes – frequently deteriorate further without appropriate management. Firstly, the primary health care system is a critical setting for individual-focused prevention and for reducing future healthcare costs. Secondly, preventive strategies at the population level can help to conserve resources, in both the short and longer term. For example, studies of the implementation of smoke-free laws illustrate that prevention can significantly reduce hospital admissions [49-51], due to the impact of tobacco smoke as a risk-factor for coronary heart disease [52]. Smoking bans may reduce rates of premature birth, and pediatric hospital admissions for asthma, by approximately 10 percent [53]. Priority interventions for prevention, including raising taxes on tobacco and alcohol products, and sugary drinks, can moderate demand for these products, while also raising the additional revenues required to fund universal access to health services, including priority treatments and essential drugs for NCDs [54].

## Improving access to NCD treatments: legal and regulatory priorities

While there is a measure of agreement about the priorities for *preventing* NCDs and reducing their risk factors (Table 1), there is no similar consensus when it comes to the legal and governance reforms that are necessary to improve access to NCD treatments. An initial point to bear in mind is that many of the regulatory reforms intended to improve access to NCD treatments will also benefit the health system generally; unlike the priorities for NCD prevention, priorities for improving treatment of the leading NCDs are unlikely to be specific to NCDs.

In our view, the legal and governance priorities for improving access to NCD treatment are grounded in human rights law [55,56]. For example, the right to health, as recognised in Article 12 of the ICESCR [23], requires countries to take progressive, concrete steps towards ensuring the availability and accessibility of quality public health and health care services, and essential drugs, especially for socially disadvantaged and marginalised groups that are vulnerable to exclusion from existing services [55]. Countries have an immediate obligation to take steps towards the full realisation of the right to health, including through legislative, administrative and budgetary measures. Article 14 of the *Framework Convention on Tobacco Control* also recognises the importance of treatment, imposing obligations on signatory countries to take effective measures to “promote cessation of tobacco use and adequate treatment for tobacco dependence” [14].

Cost-effective interventions for the primary prevention and treatment of NCDs that have been identified include multi-drug regimes for treating hypertension, glycaemic control for diabetes, and screening for cervical cancer [4,34,57,58]. On the other hand, rates of use of proven secondary prevention drugs remain shockingly low. Yusuf and colleagues found that 58% of those with coronary heart disease, and 50% of those with stroke, received none of four effective drugs for secondary prevention. In low-income countries, 83% lacked access [59]. Although people with NCDs frequently require a combination of drugs, in many low- and middle-income countries the cost of accessing these drugs pushes large segments of the population into poverty [60-62].

As noted above, improving access to cost-effective treatments for NCDs cannot be considered in isolation, but depends on improvements in the overall functioning of national health systems [4,57,63,64]. While there is no universally applicable model for reform, law and governance play an important role in strengthening the components of the primary health care system. Major components include:

• financing mechanisms for the provision of health services;

• the training, development and retention of the health workforce;

• strategies for achieving universal access to essential medicines, vaccines and technology;

• the development of an effective health information system;

• the management of infrastructure and capital investments;

• mechanisms for governance and accountability, and

• high-level leadership [65].

In Table 2, we have proposed a set of legal and regulatory priorities that countries may need to address in the course of strengthening their health systems to provide for universal access to (and financing for) essential health services and medicines, to improve the training and retention of the health workforce, and to improve national health information systems. These reforms cover a wide area. Below, we focus more specifically on law’s role in supporting access to essential health care services and medicines.

**Table 2:** Treatment: some proposed legal and regulatory priorities for improving access to treatment for the leading NCDs

Universal access to essential health services and medicines	● Formal legal recognition of the right of all members of the population to access a minimum basket of essential health care services, essential drugs, and essential technologies, at prices they can afford, on basis of medical need. This legal entitlement provides a foundation for planning and budgeting by governments, and a legal pathway for disadvantaged groups to challenge discrimination and the denial of basic health rights.
	● Protection from discrimination in access to health services: under the ICESCR, countries have an immediate obligation to respect the right to health by preventing discrimination in access to curative, palliative and preventive health services. To protect vulnerable individuals and groups from being excluded, complaints mechanisms should exist for investigating and remedying discriminatory practices, on grounds including: race, colour, caste or social status, sex, language, religious or political opinion, national origin, physical or mental disability, health status (including HIV status) and sexual orientation.
Health financing for the provision of health care services	● Effective implementation of the right to access essential health services will require governments to formally define the parameters of safety nets and health care entitlements under publicly provided and publicly subsidised schemes.
	● Governments should develop a national list of essential medicines and technologies that are available in primary health care centres and/or district hospitals. Governments can use their power as purchaser or subsidiser of medicines to negotiate lower prices.
	● Governments should also consider establishing a national procurement authority with responsibility for monitoring prices, encouraging the use of generics, reducing waste and inappropriate prescribing practices, and reducing duties, taxes and other mark-ups on essential drugs.
	● Governments should ensure that national patent laws authorise the use of the flexibilities recognised in the Agreement on Trade Related Aspects of Intellectual Property (“TRIPS”), and avoid entering bilateral agreements that exclude their right to use these flexibilities.
	● Governments should consider establishing a competition regulator to encourage competition and enforce competition laws in the health sector, in order to reduce overall costs, and to fight corruption and collusion.
Training and retention of the health workforce	● The compulsory licensing of medical and allied health professionals enables authorities to prescribe the training and qualifications required for practice, to prescribe ongoing professional training, to monitor quality and to improve accountability. The administering agency or body may also investigate complaints and impose conditions on practice.
	● Governments should investigate performance-based payment systems to encourage community-based healthcare clinics and posts to reach out to local communities, and to follow-up and manage health risks within their population.
	● Laws authorizing non-physician prescribing could increase access to drugs for chronic conditions and improve pain relief.
	● All countries should implement the *WHO Global Code of Practice on International Recruitment of Health Personnel* (2010). Countries suffering high levels of migration of domestically-trained health care workers may consider worker retention strategies, including compulsory service requirements and financial incentives.
Development of an effective health information system	● Building on a system for registration of all births and deaths, countries should implement compulsory reporting requirements for designated communicable diseases and privacy protection for health information and medical records.
	● Legislation may create a mandate for the collection and protection of a minimum national data set (comprising census data, civil registration data, notifiable diseases data, household survey data and medical records data) administered by a health information authority.

Legal recognition of the right of all members of the population to access essential health services, vaccinations, essential medicines and technologies – at prices they can afford – provides an important foundation for health resource allocation and planning by governments. Recognition of such a right would not require government to become the sole provider of these services. However, it would commit government to pursuing those intermediate objectives that will help to achieve the broader goal of universal health coverage. These intermediate objectives include: improving financial protection, improving the quality of services, and reducing the gap between the need for services and the use of those services [66].

Around two-thirds of countries have constitutions that recognize a right to health or to healthcare services [67]. In countries where this right is enforceable through the courts, it may provide a pathway for disadvantaged and vulnerable groups to challenge the denial of basic health rights. In Colombia, the Ministry of Social Protection initiated a sweeping reform of its health system – including changes in the coverage of health care services – following a finding by the Constitutional Court that systemic problems with the public health system constituted failure to fulfil the right to health [68]. Venezuela and Peru have also adjusted public health spending following court rulings [69,70].

Constitutional recognition of the right to health provides a legal basis for advocacy on behalf of vulnerable and disadvantaged groups who have been excluded from health services and from other resources for a healthy life, due to problems with cost, poor service quality, the physical location of services, and discrimination. In some cases, the constitutional right has also been recognised in legislation. For example, South Africa’s *National Health Act* provides legislative recognition of a range of health-related rights, including a commitment to progressively realise the right to health care that also appears in the constitution (Appendix A). However, even in countries where there is no constitutional right to access health services, legislative recognition of the right to health and of the state’s commitment to improving access to health services provides an important platform for advancing the interests of vulnerable populations.

In order to become a reality, the right to access health services and essential medicines needs to be implemented through government policies, programs and budgets. This depends upon several things. Firstly, governments will need to define individuals’ entitlements by formally stating the parameters of government safety nets and program entitlements, including any means-tested co-payments (contributions by individuals to the costs of their care), and right-of-access to publicly-funded services for those who cannot afford to pay. Pre-payment mechanisms, such as taxation or compulsory insurance contributions, are most effective as forms of financing and will be an indispensable part of the strategy for any country committed to fulfilling the right to health [54]. Subsidies are a feature of all financially stable financing systems that aim to provide universal coverage and to avoid imposing catastrophic financial burdens on individuals when illness strikes. Subsidies may be paid by governments, employers, by insured persons on higher incomes, or frequently a combination of these. The WHO has recommended that governments increase the public funds that are available for health financing by improving revenue collection, and prioritising national budgets so that at least 15% is appropriated to health [54]. Law can support these policies by eliminating some of the indirect barriers to accessing health care services; for example, by formalising a worker’s entitlement to a minimum number of paid days off work due to illness.

Secondly, to improve access to health services, governments need to ensure that individuals are protected from discrimination, both in their access to health services, and in the quality, appropriateness and timely delivery of those services. Under the ICESCR, countries have an immediate obligation to respect the right to health by preventing discrimination in access to curative, palliative and preventive services [55]. Although HIV-related stigma and discrimination are well recognised, obesity, diabetes, alcoholism, and increasingly, tobacco use and nicotine addiction are also stigmatised in different contexts (including in the context of healthcare provision) [71]. The 2011 Global Diabetes Plan of the International Diabetes Federation includes stopping discrimination as one of its three objectives, along with prevention and treatment of diabetes [20]. All three goals are closely linked. For example, in northern India, there are dramatic differences in rates of diagnosis and in the level of management of Type 1 diabetes in girls, as compared to boys, due to gender-based discrimination and the stigma associated with Type 1 diabetes, particularly among girls [72].

Regardless of whether protection from discrimination is a constitutional right, or is reflected in legislation, effective protection requires individuals and advocacy groups to have access to the courts or to other agencies that have both a legal mandate to investigate complaints, and the power to make determinations that bind service providers.

Thirdly, providing universal access to essential medicines at affordable prices is a necessary component of providing universal access to health care services for NCDs and other diseases [57]. The WHO has encouraged countries to amend their national legislation to formalise this right [73]. According to the WHO, three of the ten leading causes of inefficiency in health systems relate to medicines. These include the underuse of generics, the use of substandard and counterfeit medicines, and inappropriate and ineffective use of medicines [54]. In low- and middle-income countries, medicines are estimated to account for in excess of 20-30% of health spending [54]. In view of the anticipated increased demand for NCD treatments, the need to deal with these leakages, including through regulation, will become even more urgent.

By itself, legislation is not a solution for weak health systems, including the problems caused by under-budgeting, poor demand forecasting, ineffective procurement and poor distribution systems. However, governance reform can help to provide the structures and principles for improving access; for example, by extending government-funded insurance schemes to include reimbursement for essential medicines (thereby reducing the largest component of out-of-pocket household health expenditures), and by requiring the use of generic medicines, where suitable, in policies for public procurement of essential medicines [74]. Priorities for reducing the overall cost of essential medicines include exempting medicines from import duties and value added taxes, prescribing maximum mark-ups in the supply chain, and encouraging prescribers to use generics. Countries may also wish to consider the benefits of establishing a non-departmental agency with a mandate to establish a national list of essential medicines, together with guidelines for their rational use, and to monitor both the demand for these medicines and prices paid. We have proposed a number of additional potential regulatory functions of a national medicines administration (Appendix B).

The WTO Agreement on Trade-Related Aspects of Intellectual Property Rights (“TRIPS”) recognises a range of strategies (“flexibilities”) to permit countries to achieve access to generic formulations of medicines patented under law [75]. In order to take advantage of these, countries may wish to ensure that their domestic patent laws authorise the full use of TRIPS flexibilities, including the issuing of compulsory licenses and parallel importation [76-79]. National governments should be wary of surrendering TRIPS rights and flexibilities in free trade agreements, and of passing domestic legislation granting more generous patent protections on pharmaceuticals than TRIPS requires.

In the Political Declaration of the UNGA High-level Meeting, UN members committed to “comprehensive and cost-effective prevention, treatment and care for the integrated management of non-communicable diseases”, including “increased access to affordable, safe, effective and quality medicines and diagnostics”, and full use of TRIPS flexibilities [80]. Despite this rhetoric, trade disputes over access to NCD drugs are likely to increase, given the reality that US and European multinational drug companies have invested heavily in drugs for chronic conditions and see “emerging markets” (i.e. countries such as India, China and Brazil) as growth areas [81,82]. Pooled procurement and other access strategies will be required to improve access to drugs that remain unavailable at affordable prices in many countries [81]. An important impediment to treatment is that pharmaceutical companies price their drugs differently on a country-by-country basis, rather than by the capacity of the patient, or health insurer, to pay. In countries that have a substantial and growing middle class, drug companies are unlikely to want to negotiate on price – yet the majority of the world’s poor now live in middle-income countries [83].

Provision of adequate pain relief and palliative care is an important priority in the treatment of NCDs, yet the WHO estimates that approximately 80% of the global population live in countries without access to morphine for pain relief [84]. The right to adequate pain relief is implied within the right to health [85,86]. Support is growing for formal recognition of the obligation of governments to establish laws and policies to promote access to fully adequate pain management [87]. Priorities for countries include eliminating regulations that create direct or indirect obstacles to the prescription, possession and administration of opioids (including morphine) for the purposes of pain management and palliative care, in accordance with clinical need and standards of professional practice [84,88].

## Challenges to the effective use of law in response to NCDs

To summarise so far: there is a well-defined set of “best buys” for preventing NCDs at the population level. Many of these interventions rely on legislation and regulatory changes for implementation (Table 1). Law also has an important role in strengthening healthcare systems in low- and middle-income countries in order to improve NCD treatment (Table 2). A related challenge is addressing the social impact of illness for those with NCDs: this includes providing legal protection from discrimination, and effective disability support. Law reform in these areas presents a formidable challenge. Progress is likely to lag behind expectations, even in clearly defined areas such as implementation of the FCTC. For example, WHO reported in 2013 that one third of the world’s population, or 2.3 billion people, were now covered by at least one tobacco control measure recommended by the FCTC, such as warning labels, smoke-free environmental controls, tobacco taxes or tobacco advertising bans. However, only 1 billion people were covered by two or more such measures [89]. Four challenges stand out.

Firstly, although cost-effective interventions for prevention and treatment do exist, demands for funding them – to the tune of $11.4 billion per year, mostly for treatment [34] – have come during a sustained global economic downturn. Funding on this scale is unlikely to materialise [90]. Delivering treatment reliably and equitably depends on strengthening primary healthcare systems. Progress tends to be slow, partly because of the need for investment in all elements of the system (financing mechanisms, essential medicines, health workforce, and so on), and for effective integration between them. In many low- and middle-income countries, structures and programs have already been established for the provision of HIV-related treatment. Given the similarities between HIV and NCDs – including the need for continuity of care in an environment of health system constraints – building on these existing structures may, particularly in countries with severely limited resources, provide the most effective way of meeting growing demand for NCD services [28,91].

Secondly, NCDs are exacerbated by the global marketing of tobacco, alcohol and unhealthy food [92,93]. Despite their differences, tobacco, alcohol and food manufacturers and retailers all share an economic interest in expanding markets for the risk factors that are driving the global epidemic of NCDs [94-97]. Each of the evidence-based, cost-effective measures summarised in Table 1 represent a potential threat to revenues. As such, industry organisations will perceive them as a *business risk* and will seek to manage them through political lobbying, legal manoeuvres, pre-emptive self-regulatory schemes, corporate social responsibility initiatives, stakeholder marketing [98], advertising, and other strategies for shaping favourable community perceptions. In debates about business regulation, rhetoric plays a huge part. For example, it is highly ironic that even as multinational tobacco companies rush to exploit huge markets and weak tobacco controls in developing countries, evidence-based international standards such as the FCTC are framed, by some, as an instrument of neo-colonialism that threatens national sovereignty [99]. To a significant degree, progress will depend on the capacity and willingness of governments – spurred on by civil society organisations – to regulate the business sector.

The role of civil society deserves special emphasis. The capacity for full engagement by civil society requires respect for freedom of speech and freedom of association. Patients’ rights groups, medical and public health associations, organisations promoting the rights of women, children and disabled people, and legal and human rights organisations must have the freedom to advocate for the government actions that are necessary to address NCDs.

The influence of industry lobbyists is only one reason why governments may be reluctant to introduce priority interventions for NCDs. Other reasons include the relatively muted *demand* for these measures in countries where a large proportion of the population has low levels of education, and the reluctance of governments to adopt measures that could be perceived as creating a less favourable environment for foreign investment. In addition, in many countries, public health laws are outdated, technical capacity is lacking, the rule of law is weak, conflicts of interest are common, and corruption is part of the political reality. National governments must also navigate WTO rules and obligations under multilateral agreements and (“BITs”), while resisting pressure from high-income countries – including their own development partners – whose domiciled companies benefit from global trade and investment in tobacco, alcohol and unhealthy food [36-38,100,101]. Innovator governments are likely to be singled out for special treatment, as illustrated by the BIT dispute between Philip Morris and Uruguay over the latter’s proposed tobacco health warning legislation [102], and the suite of challenges to Australia’s legislation requiring plain packaging of tobacco products [103]. Within this challenging environment, the implementation of effective measures to reduce risk factors for NCDs is more difficult than many assume.

Thirdly, in many high-income countries, resistance to the implementation of preventive measures appeals to libertarian values and to the rhetorical force of concepts such as “personal responsibility” and distaste for a “nanny state” [104-111]. Nevertheless, the priority recommendations set out in Table 1 involve little interference with the freedom of adults to make their own choices, with the exception of smoke-free laws and drink-driving controls, which also aim to prevent harm to others. Rather, these interventions constrain the for-profit activities of manufacturers and retailers whose products make a significant contribution to the burden of disease. It is true that taxes increase the cost of choosing unhealthy products, and that advertising controls moderate exposure to marketing of unhealthy products [97]. Yet, governments have a legal obligation, as stewards of the health of their populations, to create conditions for the realisation of the highest attainable standard of health [55]. Governments should not abandon this responsibility to private markets.

A final challenge worth noting, as Table 1 illustrates, is that since NCDs are attributable to a wide range of risk factors, an effective response will likely rest on the combined impact of a variety of interventions, each of which is likely to face vigorous resistance from industry. Reducing tobacco use, for example, requires at a minimum that countries raise excise taxes, eliminate smoking in public places, impose health warnings and point-of-sale controls, and comprehensively ban the advertising, promotion and sponsoring of tobacco products. Rapid implementation of the full set of interventions may be difficult to achieve: reform of public health laws tends to be incremental.

Three important practical considerations follow from these obstacles to the implementation of effective laws and policies for NCD prevention and treatment. First, progress requires high-level leadership by national political figures. Presidents, Prime Ministers and senior cabinet members need to become personally involved: their leadership should be directed towards framing NCDs as an obstacle to national economic and social development, and to fostering an inter-sectoral, government-wide response [41]. Secondly, NCDs call for an all-of-government response not only because many of the priority interventions will be implemented outside of the health sector, but also because broadly-based political support will be needed to secure passage of the necessary laws and budgets. In some countries, existing multi-sectoral structures for coordinating a national response to HIV may be appropriate for adapting to NCDs [91].

Thirdly, governments should focus their initial efforts and budgets on those measures that will yield the greatest results in terms of reducing death and disability. Countries with high rates of tobacco use cannot hope to make significant progress in reducing NCDs until they make tobacco control a priority [112,113]. Countries that have not yet signed the FCTC should do so urgently: this includes high-population countries such as Indonesia, where 61% of males are current smokers and where smoking rates for boys aged 15–19 doubled from 14% to 33% between 1995 and 2004 [114,115].

Within the area of tobacco control, the WHO’s MPOWER package selects six priority areas for policy action from the FCTC and explains their rationale and evidence base [116]. One study estimates that if MPOWER policies had been implemented globally in 2010, smoking rates would have fallen to 13.2% by 2030 (523 million smokers), instead of the 22% (872 million smokers) estimated on current trends [117]. The *Lancet* NCD Action group has argued that countries with limited human and financial resources should prioritise two highly cost-effective population-wide strategies (tobacco control, and dietary salt reduction), and one individual-based strategy (treatment of individuals at high risk for CVD or who have had a heart attack or stroke) [21,118]. Once these interventions are in place, countries should expand their response as resources permit to include other cost-effective interventions, particularly those identified in Table 1. The good news is that governments implementing these priority measures can expect rapid – rather than gradual – reductions in mortality, particularly from cardiovascular disease [119].

## Establishing a national legislative and governance framework for NCDs

There is a growing literature that aims to assist countries to identify policy priorities for NCDs and to make best use of limited resources [4,5,10,12,13,21,120-122]. Not all priorities require legislation, and the mechanism for implementing priority measures will vary from country to country. Relevant pathways for implementation include the direct use of Presidential powers, legislation passed by Parliament, and regulations issued by executive agencies exercising delegated powers under relevant codes or statutes. Two important variables that are relevant to implementation are the constitutional structure and division of legislative powers between national and regional parliaments; and secondly, the presence or absence of explicitly-recognised fundamental rights and freedoms within federal and state constitutions.

In countries that have a federal structure, legislative powers will be shared between national and state (i.e. regional) governments, and each will have defined responsibilities. Each government will also have its own budget, and sources of revenue. National laws, effectively enforced, have the power to improve the health of the entire country, although their passage may be more difficult to achieve in political terms. On the other hand, provided there is adequate political leadership, state and local governments may have considerable flexibility to experiment with innovative approaches that have not yet achieved widespread political support, and to build the evidence base for effective public health law [123]. Where appropriate, public health advocates should encourage “competitive federalism”, by encouraging regional and city leaders to implement best practice, and to be national and international leaders in health policies.

National constitutions provide both constraints and opportunities for implementing priorities for the control of NCDs. In the United States, for example, the First Amendment to the Constitution constrains the capacity of legislatures to pass legislation prohibiting or limiting the truthful advertising of tobacco and alcohol products to adults [124,125]. First Amendment jurisprudence suggests that it will be very difficult for public health laws forbidding retail advertising, suppressing tobacco trademarks or other distinguishing brand features, or imposing mandatory warnings on products, to pass constitutional scrutiny in the absence of a substantial body of evidence demonstrating the independent impact of each one of these laws in reducing smoking rates or other harmful exposures. Countries that prioritise freedom of speech over other public interests are likely to be followers, rather than leaders, when it comes to laws that seek to re-balance the informational environment in favour of public health [126,127].

On the other hand, in countries whose constitutions give individuals a right to challenge laws that infringe constitutionally protected rights, or to challenge the failure of governments to fulfil or protect those rights, there are opportunities for litigants to seek protection from harmful exposures and for access to other determinants of a healthy life [128]. In one well-known example, the Supreme Court of India held that smoking in a range of public places breached the constitutional right to protection of life and personal liberty [129]. The Court called upon federal and state governments to pass legislation prohibiting smoking in a range of public settings. These restrictions were included in subsequent federal tobacco control legislation passed in 2003 [130]. This example also illustrates the importance of litigation as a strategy for responding to industries that promote harmful products [14,131].

In this section, we highlight two important steps in the practical process of establishing legislative and governance frameworks for a national response to NCDs. The first step is to review current laws, policies and programs in order to assess the state of progress, and to map where responsibility lies for these functions within government. In some countries, this process will identify clear gaps in a country’s laws and regulatory capacity. Based on this assessment, national leaders should make public and time-bound commitments to address gaps and to implement measures that address the most important health challenges the country is facing. These commitments should relate to the current political cycle. When introducing and debating legislation, governments may find it useful to refer to the obligations they owe under international law to implement the provisions of international agreements such as the FCTC [14], and to respect, protect and fulfil the right to health, which – as described above – also enjoys constitutional protection in many countries [67].

Passing new laws can be difficult, and health leaders should also consider whether government agencies could make better use of powers that are already available. For example, in Australia, the competition and consumer regulator put an end to the marketing of so-called “light” and “mild” cigarettes by accepting enforceable undertakings from tobacco manufacturers in exchange for discontinuing court actions against them for misleading and deceptive conduct under consumer protection legislation [132]. When passing legislation or making regulations, governments should ensure that public health officials have a clear mandate, clear enforcement powers, and adequate resources for discharging their public health responsibilities.

A second step for governments is to create an enabling institutional and governance environment to drive necessary reforms. This includes governance reforms within the health portfolio, as well as inter-sectoral reforms that take account of the interests of different ministries and create the capacity for a government-wide response [39,40,133].

Within the health portfolio, countries need – at a minimum – a dedicated tobacco control unit that is free from interference from the tobacco industry and tobacco growers, with secure funding, adequate human resources, enforcement powers, and geographical reach. A number of countries have successfully introduced health promotion agencies, funded by tobacco taxes, to lead research, information dissemination, and social marketing efforts [134]. The WHO has repeatedly emphasised that countries can achieve sustainable financing for their infrastructure requirements by raising taxes on tobacco and alcohol [4,11,54]. Taxes on foods that are high in sugar, salt and fat, and on sugar-sweetened beverages, may provide additional revenues [4,135-137].

The priorities for reducing risk factors for NCDs require the involvement of a range of ministries outside health. In practical terms, this will require governance structures to ensure policy coherence and to formalise and maintain a government-wide commitment. Cross-ministerial executive committees and taskforces, and cross-government strategies setting out the contributions expected from each Ministry towards shared goals, may be useful and should be adapted to local circumstances. In Mexico, the National Council for the Prevention and Control of Chronic, Non-communicable Diseases was established by Presidential decree. The Council acts as the permanent coordinating body for national action on NCDs, coordinating actions among federal government agencies, and between federal and State governments under the National Health Council [138]. In the United States, President Barack Obama established the National Prevention Council, which now comprises heads of 20 federal agencies [139,140]. The Council was required to develop a national prevention strategy and to provide annual progress reports on the actions federal agencies should take to help achieve national goals for reductions in tobacco use, sedentary behaviour, and poor nutrition [141].

National responses to HIV provide a useful comparator for responding to NCDs. In many developing countries, governments established national AIDS commissions outside of the ministry of health in order to bring together the ministries of health, labour, education, transport, and justice, and to design and implement national AIDS strategies that engaged all relevant sectors. Similarly, collaboration between international development partners was facilitated by UN Theme Groups on HIV/AIDS in each country [142]. These were convened by the UN Resident Representative and often the chair was rotated among the participating agencies working in that country. In some cases their membership was expanded to include non-UN agencies, government representatives, and leading civil society organisations, including representatives of people living with HIV [143].

## Accountability for progress in law and governance reform for NCDs

Global architecture for the response to NCDs continues to evolve. There have been calls for the development of a framework convention for the control of alcohol [144], and of obesity [145], and considerable momentum for a framework convention on global health to provide a unifying framework for global health efforts [146]. NCDs were not included in the Millennium Development Goals (“MDGs”) but have been recognised as a major item on the agenda for sustainable development and poverty reduction [147], and more recently, included in a set of draft development goals for the post-2015 era [148].

The Political Declaration from the UNGA High-level Meeting identified the WHO as the international agency with primary responsibility for leading the global response to NCDs, in coordination with other UN and international agencies [80]. The WHO’s primary strategy document is the *Global Action Plan for the Prevention and Control of Noncommunicable Diseases 2013–2020*, adopted by the World Health Assembly (“WHA”) in May 2013 [149]. As recommended by the WHA, the United Nations Economic and Social Council (“ECOSOC”) has established a United Nations Task Force on NCDs, co-chaired by WHO, which will coordinate the activities of UN organisations and intergovernmental organisations in implementing the Action Plan [149-152]. This Task Force will also incorporate the work of the United Nations Ad Hoc Interagency Task Force on Tobacco Control [153]. WHO has prepared a draft division of roles and responsibilities, in consultation with the Task Force members [154]. Separately, the WHA has requested the Director-General to develop draft terms of reference for a separate “global coordination mechanism” to facilitate engagement among a wider group of stakeholders including Member States, UN funds, programmes and agencies, and NGO and private sector entities, while protecting public health from the potential for conflicts of interest [155,156].

Key to the success of these global structures will be their capacity to harness and integrate the energy of civil society organisations and to create a global movement that influences the policy agenda at country level. Unlike the Global Fund [157] and other large global partnerships with formalised structures that are organised around the distribution of funding, the global coordinating mechanisms for NCDs will not primarily function as funders. Despite their impact on global health and economic development, there remains a dramatic disconnect between the impact of NCDs and the resources available to respond to them: in 2010 only 0.8% of total development assistance in health (US$18.2 million) was directed to NCDs [81,158]. In the short term at least, NCDs will need to be addressed predominantly with domestic funding [159]. At the global level, economic incentives, legal obligations assumed under international law, and political commitment (formalised through goals, targets, declarations and partnership structures) can all help to generate greater momentum for policy change at the country level [160,161]. It seems clear, however, that the global process that underpins the emerging architecture for NCDs is largely a political one. Through formal meetings of member states, consultation processes and strategic documents, national governments are being encouraged to provide the funding, to develop the multisectoral structures, and to make the legislative, governance and health systems reforms that are required to fully implement the Global Action Plan.

The comprehensive global monitoring framework, adopted by the WHA in May 2013, represents one possible tool for strengthening the commitment of national governments to prioritise NCDs [162]. Building on the “25 by 25” goal adopted in 2012 [19], the monitoring framework consists of nine voluntary global targets for addressing specific behavioural and biological risk factors, and 25 indicators for measuring progress towards each target [162]. The voluntary targets include a 10% relative reduction in the harmful use of alcohol, a 30% reduction in current tobacco use by persons aged 15 years and above, a 30% reduction in mean population salt intake, and a halt in the rise of obesity. Reports on progress will be submitted to the WHA in 2015, 2020, and finally in 2025 [162].

A striking feature of the monitoring framework is that it focuses predominantly on monitoring behavioural risk factors and physiological and epidemiological outcomes, rather than accountability for implementation of the evidence-based interventions identified prior to the UNGA High-level Meeting, and again in the Global Action Plan (Table 1). The availability of generic essential medicines and technologies for NCDs, together with access to opioid analgesics, are appropriately included as indicators for the health system's response. Additional indicators also make reference to vaccination for hepatitis B and human papilloma virus, eliminating industrially-produced *trans* fats from food, and policies to reduce the marketing of foods high in saturated fats, salt and free sugars to children. On the other hand, the monitoring framework imposes no accountability for implementing WHO’s recommended cost-effective interventions for reducing tobacco use and harmful use of alcohol, and for improving diets. The risk is that the framework will prove more useful for its awareness-raising role and capacity to generate data about the problem, rather than its capacity to hold countries accountable for implementing the most powerful solutions. To put it bluntly, what gets measured gets done: what is not measured is likely to rank as a lower priority by governments.

In 2013, following the adoption of the Global Action Plan 2013–2020, WHO initiated a separate consultation on the indicators to inform on progress in implementing the action plan [163]. The WHO’s survey instrument for assessing national capacity and response to NCDs (the “NCD Country Capacity Survey Tool”), has a greater focus on policy indicators, including whether or not countries are implementing taxes on tobacco, alcohol and foods high in sugar or fat, and whether legislation has been adopted to regulate food and beverage marketing to children, promote breastfeeding, limit *trans* fats and reduce salt consumption [164]. However, this instrument is also unlikely to provide adequate data on the full extent to which policies have been operationalized, including through legislation and regulation. It will likely be left to civil society networks – including, for example, the recently-announced INFORMAS network for monitoring the food environment and obesity – to fill the gap by documenting the extent to which countries are actually implementing the laws and policies that can be expected to reduce NCD risk factors over time [165,166].

The global monitoring and reporting framework [167,168] that has evolved to monitor implementation of the Declaration of Commitment made at the 2001 UN General Assembly Special Session on HIV/AIDS [169], and subsequent Political Declarations [170], holds important lessons for NCDs. For example, monitoring frameworks should include specific legal and policy indicators, rather than being predominantly focused on changes in epidemiology or access to treatment. Under the Global AIDS Response Progress Reporting system, countries are requested to report biennially on progress in implementing national-level HIV policies, strategies and laws through the National Commitments and Policy Instrument [171]. This instrument not only measures progress in implementing priority laws and policies, but also enables civil society organisations and development partners to comment on the rate of progress and what remains to be done [172].

## Priorities for development assistance for NCD law and governance reform

In earlier sections of the paper we argued that although progress in preventing and treating NCDs will require the substantial use of legal and regulatory powers by governments, there are many obstacles to this, and inadequate accountability through the global monitoring framework. At the same time, an analysis of WHO Country Cooperation Strategies in 2012 found that the number of requests for technical assistance in the area of NCDs exceeded the number of requests for assistance in health systems strengthening, and communicable diseases, respectively [173]. Demand for *legal* technical assistance is likely to become increasingly important as countries begin to seriously consider the most appropriate interventions for both prevention and treatment. In light of this, what are the most realistic steps that can be taken to promote the role of law and to better support NCD-related law reform at the country level?

Legal and regulatory strategies for the prevention and treatment of NCDs are not self-executing, but require adherence to the rule of law, and a trained public health workforce that is resistant to corruption. Investing in prevention means improving institutional capacity to implement and enforce taxation laws, advertising bans and other controls on business conduct. It requires an effective customs service and police force capable of investigating and prosecuting offences for smuggled and untaxed goods, such as alcohol and tobacco products. It requires a functioning court system for prosecuting offenders and imposing penalties. In the case of legal and regulatory efforts to improve diets and to reduce obesity, it requires the capacity to articulate policy goals and to lead partnerships with the private sector in ways that strengthen, rather than undermine, progress towards national health goals. As these examples illustrate, development assistance to implement priority interventions for NCDs is likely to involve collaboration with a range of ministries, including health, justice, finance and education.

Given these complexities, we identify three priorities for the provision of technical assistance to assist countries with the legal and governance reforms that are needed to implement the WHO’s Global Action Plan on NCDs [174]. Firstly, countries need high-quality legal resources (handbooks, manuals, tool kits) to assist them to evaluate reform options, taking into account the unique legal context of each country and providing examples of best practice in low- and middle-income countries. Public health journals routinely call for leadership and policy change, yet are usually silent about the governance and law reform processes that are necessary to make these changes happen. With its unique mandate in public health law, the WHO has an important opportunity to move beyond its two major achievements in public health law – the FCTC and International Health Regulations – and to share examples of laws that have been used effectively to implement its own recommendations for NCD prevention and treatment [175].

Secondly, investment in capacity-building is urgently needed to nurture future leaders in public health law, governance and advocacy. Training, including the sharing of experience and technical information, would be especially useful on a regional basis, between countries that share a common language, similar legal structure and/or regional outlook. An understanding of the role of public health law is important not only for trained lawyers, but for other leaders in government, Ministry officials, and civil society representatives. Both of the priorities above will require funding – remarkably modest funding, in comparison to the significant sums required for access to NCD treatments. The value of legal assistance in health development is poorly understood by donors and development agencies, despite the significant profile that law and governance reform has in NCD control, and country requests for assistance. In its absence, it is not surprising that business interests – including tobacco, alcohol and food companies – are seeking to fill the policy vacuum with weak, self-regulatory regimes that impose few constraints on their behaviour [176-181].

Thirdly, leadership in public health law is needed at the global level. Despite its strong constitutional mandate and comparative advantage as a trusted source of technical expertise, the WHO currently lacks capacity to discharge this role effectively. Nevertheless, under the evolving division of responsibilities between international organisations making up the UN Interagency Task Force on NCDs, it is likely that WHO and the United Nations Development Program (“UNDP”) will be the lead agencies addressing matters relating to law, gender, and human rights [150]. However, significant opportunities also exist for other organisations to promote the role of public health law. The World Bank, regional development banks, large bilateral funders and other UN agencies can contract legal expertise as required, yet none appear to have yet recognised the scale of need that would arise if countries seek to implement the Global Action Plan in the spirit that advocates are hoping for. Other organisations, including the International Development Organization (“IDLO”), the International Parliamentary Union, and the Commonwealth Secretariat, could also play a useful role. Academic networks represent an untapped resource, but need to be linked to development agencies and country needs in a more systematic and productive way. Important work is being done through the Bloomberg Foundation-funded International Legal Consortium [131], which aims to support tobacco control initiatives in high-burden countries. The Framework Convention Alliance (the peak civil society organisation supporting the FCTC) [182] and regional organisations advocate for law reform, monitor progress, and produce valuable research. However, the challenge remains for an appropriate agency or organisation, in partnership with WHO and others, to assume global leadership in the legal and regulatory aspects of NCD prevention and control.

## Conclusion

This paper has identified priority areas for law reform addressing the prevention, treatment, and social consequences of NCDs. The priorities for prevention and treatment of NCDs, summarised in Tables 1 and 2, illustrate that legal and regulatory reforms lie at the heart of a successful national response to NCDs and that law’s role is more significant than has been acknowledged. At the same time, these reforms are not self-executing and will be challenging to achieve, including population-focused measures to reduce tobacco use, obesity, the over-consumption of alcohol, and dietary imbalances. Stricter controls on business, in order to align profit-motivated activities more closely with public health goals, are essential but will be vigorously contested. Countries will need a robust sense of their own health sovereignty to implement the reforms that are needed. The priority for low- and middle-income countries should be the proven, evidence-informed “best buys”, that experience already shows will make the most difference to the burden of disease (Table 1).

Leadership and accountability are vital requirements for the success of national efforts to prevent and control NCDs [5]. Globally, the emerging governance structures rely fundamentally on national governments to assume leadership and to implement effective laws and policies. Countries are not being invited to assume legal obligations under a framework convention, nor is there (yet) any global financing mechanism for NCDs. The processes for integrating the voice of civil society also remain ambiguous. However, it is these very features that have motivated calls for a Framework Convention on Global Health to replace the Millennium Development Goals [146]. The proposed global monitoring framework for NCDs must be strengthened to include comprehensive monitoring, reporting and accountability for legal and policy action by governments. The experience of HIV has shown the value of a comprehensive, transparent global monitoring framework that includes indicators for policy, law and human rights.

Global leadership in public health law is currently weak and fragmented. No global agency combines resources, technical capacity, and a clear mandate in this area. WHO maintains a database of health legislation, but this is neither complete nor available in electronic format. As Attaran and colleagues point out, “without reliable sources, good laws go unnoticed” [175]. Also missing are sources that elaborate on the history, context and process of passing good and effective public health laws. The near invisibility of success stories means that it is more difficult for low- and middle-income countries and governments to adopt best practice and to benefit through shared learning. The challenge remains for development agencies to recognise the growing need for legal assistance – especially with respect to NCDs – and to devote more resources to this area.

## Appendix A

### Constitution of the Republic of South Africa, Section 27: the right to health care, food, water and social protection

1. Everyone has the right to have access to

a. health care services, including reproductive health care;

b. sufficient food and water; and

c. social security, including, if they are unable to support themselves and their dependants, appropriate social assistance.

2. The state must take reasonable legislative and other measures, within its available resources, to achieve the progressive realisation of each of these rights.

3. No one may be refused emergency medical treatment.

### The Goals of South Africa’s *National Health Act* (Act No. 61 of 2003)

2. Objects of Act

The objects of this Act are to regulate national health and to provide uniformity in respect of health services across the nation by:

a) Establishing a national health system which:

i) encompasses public and private providers of health services; and

ii) provides in an equitable manner the population of the Republic with the best possible health services that available resources can afford;

b) Setting out the rights and duties of health care providers, health workers, health establishments and users; and

c) Protecting, respecting, promoting and fulfilling the rights of:

i) the people of South Africa to the progressive realisation of the constitutional right of access to health care services, including reproductive health care;

ii) the people of South Africa to an environment that is not harmful to their health or well-being;

iii) children to basic nutrition and basic health care services contemplated in Section 28(l)(c) of the Constitution; and

iv) vulnerable groups such as women, children, older persons and persons with disabilities.

## Appendix B

### Some possible regulatory functions of a national medicines administration authority [74]

• Establishing a national list of essential medicines that responds to country-specific needs and disease burden, with a focus on primary care.

• Establishing evidence-based clinical guidelines for rational use of prescription medicines.

• Monitoring demand for essential medicines; monitoring prices in the public and private sectors.

• Educating prescribers and establishing financial incentives for prescribers to substitute generic brands.

• Preferential registration and quality assurance of generic medicines.

• Working with donors to reduce duplication of distribution systems.

• Investigating counterfeit medicines and referring cases to law enforcement authorities.

• Negotiating prices and licenses on behalf of government-funded or subsidised health insurance schemes.

• Monitoring safety and quality of medicines; investigating safety issues – using legal powers to inspect premises, to remove, test and recall products, and to restrain misleading and fraudulent practices.

• Negotiating prices on behalf of government-funded and -subsidised schemes

• Advising government on use of TRIPS flexibilities.

## Competing interests

Roger Magnusson:

• Financial competing interests: none

• Non-financial competing interests:

◦ The author holds a research grant from the Australian Research Council (DP120104540) entitled Evidence-informed legal strategies for preventing cancer, heart disease and diabetes: what can Australia learn from the United States?

◦ The author teaches a unit of study entitled ‘Law and Healthy Lifestyles’, which surveys legal and regulatory responses to non-communicable diseases, at Sydney Law School, Australia, and at Georgetown University Law Center in Washington DC.

◦ The author is not aware of any other non-financial interests that should be declared.

David Patterson

• Financial competing interests: DP is an employee of the International Development Law Organization (IDLO), and manages IDLO’s health program. Increased funding for the global response to legal aspects of NCDs as a result of this article may result in increased funds for IDLO’s health law program.

• Non-financial competing interests: none.

## Authors’ contributions

RM prepared the first draft and guided preparation of the manuscript. DP contributed advice and text to the initial draft, and both authors subsequently discussed, edited and contributed text to subsequent versions of the draft. Both authors read and approved the final manuscript.

## References

[B1] Lozano R Naghavi M Foreman K Lim S Shibuya K Aboyans V Abraham J Adair T Aggarwal R Ahn S AlMazroa M Alvarado M Anderson H Anderson L Andrews K Atkinson C Baddour L Barker-Collo S Bartels D Bell M Benjamin E Bennett D Bhalla K Bikbov B Abdulhak A Birbeck G Blyth F Bolliger I Boufous S Burcello C Global and regional mortality from 235 causes of death for 20 age groups in 1990 and 2010: a systematic analysis for the global burden of disease study 2010 Lancet 2012 380 2095 2128 10.1016/S0140-6736(12)61728-0 23245604 PMC10790329

[B2] Hunter D Reddy K Noncommunicable diseases N Engl J Med 2013 369 1336 1343 10.1056/NEJMra1109345 24088093

[B3] World Health Organization Noncommunicable Diseases: Country Profiles 2011 2011 Geneva: World Health Organization

[B4] World Health Organization Global Status Report on Noncommunicable Diseases 2011 Geneva: World Health Organization

[B5] Beaglehole R Bonita R Horton R Adams C Alleyne G Asaria P Baugh V Bekedam H Billo N Casswell S Cecchini M Colagiuri R Colagiuri S Collins T Ebrahim S Engelgau M Galea G Gaziano T Geneau R Haines A Hospedales J for The Lancet NCD Action Group and the NCD Alliance Priority actions for the non-communicable disease crisis Lancet 2011 377 1438 1447 10.1016/S0140-6736(11)60393-0 21474174

[B6] Lim SS Vos T Flaxman AD Danaei G Shibuya K Adair-Rohani H AlMazroa MA Amann M Anderson HR Andrews KG Aryee M Atkinson C Bacchus LJ Bahalim AN Balakrishnan K Balmes J Barker-Collo S Baxter A Bell ML Blore JD Blyth F Bonner C Borges G Bourne R Boussinesq M Brauer M Brooks P Bruce NG Brunekreef B Bryan-Hancock C A comparative risk assessment of burden of disease and injury attributable to 67 risk factors and risk factor clusters in 21 regions, 1990-2010: a systematic analysis for the Global Burden of Disease Study 2010 Lancet 2012 380 2224 2260 10.1016/S0140-6736(12)61766-8 23245609 PMC4156511

[B7] World Economic Forum and World Health Organization From Burden to “Best Buys”: Reducing the Economic Impact of non-Communicable Diseases in low- and Middle-Income Countries 2011 Geneva: World Economic Forum and World Health Organization

[B8] Sivaramakrishnan K Parker R The united nations high level meeting on the prevention and control of noncommunicable diseases: a missed opportunity? Am J Public Health 2012 102 2010 2012 10.2105/AJPH.2012.300768 22994178 PMC3477959

[B9] The World Bank The Growing Danger of non-Communicable Diseases: Acting now to Reverse Course. Conference Edition 2011 Washington DC: World Bank

[B10] The World Bank Towards a Healthy and Harmonious Life in China 2011 Washington DC: World Bank

[B11] World Health Organization Draft Action Plan for the Prevention and Control of Noncommunicable Diseases 2013–2020. A66/9, Appendix 3 (Endorsed WHA May 2013) 2013 Geneva: World Health Organization

[B12] Chisholm D Edejer TT-T Baltussen R Evans DB Ginsberg G Lauer JA Lim S Ortegon M Salomon J Stanciole A What are the priorities for prevention and control of non-communicable diseases and injuries in sub-Saharan Africa and South East Asia? BMJ 2012 344 e586 10.1136/bmj.e586 22389336

[B13] Jamison DT Frenk J Ghosh G Goldie SJ Guo Y Gupta S Horton R Kruk ME Mahmoud A Mohohlo LK Ncube M Summers LH Pablos-Mendez A Reddy KS Saxenian H Soucat A Ulltveit-Moe KH Ulltveit-Moe KH Yamey G Alleyne G Arrow KJ Berkley S Binagwaho A Bustreo F Evans D Feachem RGA Global health 2035: a world converging within a generation Lancet 2013 382 1898 1955 at 1926–1927 10.1016/S0140-6736(13)62105-4 24309475

[B14] World Health Organization WHO Framework Convention on Tobacco Control. WHA56.1 (entered into force 27 February 2005) 2003 Geneva: World Health Organization

[B15] World Health Organization Global Strategy to Reduce the Harmful Use of Alcohol WHA63/13 – Annex 2 (Endorsed WHA May 2010) 2010 Geneva: World Health Organization

[B16] World Health Organization WHO Global Strategy on Diet, Physical Activity and Health. WHA57.17. (Endorsed WHA May 2004) 2004 Geneva: World Health Organization

[B17] World Health Organization Set of Recommendations on the Marketing of Foods and non-Alcoholic Beverages to Children (WHA63.14, Endorsed WHA May 2010) 2010 Geneva: World Health Organization

[B18] World Health Organization A Framework for Implementing the set of Recommendations on the Marketing of Foods and non-Alcoholic Beverages to Children 2012 Geneva

[B19] World Health Organization 65th World Health Assembly closes with new global health measures [http://www.who.int/mediacentre/news/releases/2012/wha65_closes_20120526/en/index.html]

[B20] International Diabetes Federation Global diabetes plan 2011–2021 [http://www.idf.org/global-diabetes-plan-2011-2021] 35914061

[B21] Bonita R Hospedales J de Courten M Capewell S Beaglehole R Magnusson R Bovet P Zhao D Malta DC Geneau R Suh I Thankappan KR McKee M Lancet NCDAG, Lancet NCDAG Country actions to meet UN commitments on non-communicable diseases: a stepwise approach Lancet 2013 381 575 584 10.1016/S0140-6736(12)61993-X 23410607

[B22] World Health Organization Constitution of the World Health Organization (Preamble) 1948 Geneva: World Health Organization

[B23] UN General Assembly International Covenant on Economic, Social and Cultural Rights 1966 993 United Nations, Treaty Series 3 Article 12

[B24] Organization of African Unity (OAU) African Charter on Human and Peoples' Rights ("Banjul Charter") June 1981,?CAB/LEG/67/3 rev. 5, 21 I.L.M. 58 (1982), Article 16

[B25] Council of Europe European Social Charter (Revised) 3 May 1996,?ETS 163, Article 11

[B26] Organization of American States (OAS) Additional Protocol to the American Convention on Human Rights in the Area of Economic, Social and Cultural Rights ("Protocol of San Salvador") 16 November 1999,?A-52, articles 10-11 12344012

[B27] Constituição Federal de 1988 [Federal Constitution of 1988] Brazil

[B28] Lamptey P Dirks R Building on the AIDS response to tackle noncommunicable disease Global Heart 2012 7 67 71 10.1016/j.gheart.2012.01.010 25691169

[B29] NCD Alliance Noncommunicable diseases: a priority for women’s health and development NCD Alliance; 2011. [http://ncdalliance.org/womenandncds]

[B30] United Nations Development Program Discussion paper: addressing the social determinants of noncommunicable diseases 2013 New York

[B31] Brawley O Avoiding cancer deaths globally CA Cancer J Clin 2011 61 67 68 10.3322/caac.20108 21296854

[B32] Shaw J Sicree R Zimmet P Global estimates of the prevalence of diabetes for 2010 and 2030 Diabetes Res Clin Pract 2010 87 4 14 10.1016/j.diabres.2009.10.007 19896746

[B33] Asaria P Chisholm D Mathers C Ezzati M Beaglehole R Chronic disease prevention: health effects and financial costs of strategies to reduce salt intake and control tobacco use Lancet 2007 370 2044 2053 10.1016/S0140-6736(07)61698-5 18063027

[B34] World Health Organization Scaling up Action Against Noncommunicable Diseases: how Much Will it Cost? 2011 Geneva: World Health Organization

[B35] Cecchini M Sassi F Lauer J Lee Y Guajardo-Barron V Chisholm D Tackling of unhealthy diets, physical inactivity, and obesity: health effects and cost-effectiveness Lancet 2010 376 1775 1784 10.1016/S0140-6736(10)61514-0 21074255

[B36] McGrady B Trade and Public Health: The WTO, Tobacco, Alcohol, and Diet 2011 Cambridge: Cambridge University Press

[B37] Mitchell A Voon T Implications of the World Trade Organization in combating non-communicable diseases Public Health 2011 125 832 839 10.1016/j.puhe.2011.09.003 22030320

[B38] Voon T Flexibilities in WTO law to support tobacco control regulation Am J Law Med 2013 39 199 217 23815028 10.1177/009885881303900201

[B39] World Health Organization Discussion paper. Intersectoral action on health: a path for policy-makers to implement effective and sustainable intersectoral action on health. First global ministerial conference on healthy lifestyles and noncommunicable disease control, Moscow, 28–29 April, 2011 [http://www.who.int/nmh/publications/ncds_policy_makers_to_implement_intersectoral_action.pdf]

[B40] World Health Organization Discussion paper. Effective approaches for strengthening multisectoral action for NCDs, 19 March 2012 [http://www.who.int/nmh/events/2012/consultation_march_2012/en/index.html]

[B41] Beaglehole R Bonita R Alleyne G Horton R Li L Lincoln P for the Lancet NCD Action Group UN high-level meeting on non-communicable diseases: addressing four questions Lancet 2011 378 449 455 10.1016/S0140-6736(11)60879-9 21665266

[B42] World Health Organization Prevention and control of noncommunicable diseases: options and a timeline for strengthening and facilitating multisectoral action for the prevention and control of noncommunicable diseases through partnership: report by the Secretariat. Sixty-fifth World Health Assembly. 15 May 2012, A65/7

[B43] World Health Organization The World Health Report 2003: Shaping the Future 2003 Geneva: World Health Organization

[B44] Bayer R Private Acts, Social Consequences 1989 New York: Macmillan

[B45] HHS Panel on Antiretroviral Guidelines for Adults and Adolescents Guidelines for the use of antiretroviral agents in HIV-1-infected adults and adolescents. Department of Health and Human Services (USA), updated 12 February 2013 [http://aidsinfo.nih.gov/contentfiles/lvguidelines/adultandadolescentgl.pdf]

[B46] Smith L Ramakrishnan U Ndiaye A Haddad L Martorell R The importance of women’s status for child nutrition in developing countries; International Food Policy Research Institute, Research report 131, 2003 [http://www.ifpri.org/publication/importance-womens-status-child-nutrition-developing-countries]

[B47] Choudhury K Hanifi M Rasheed S Bhuiya A Gender inequality and severe malnutrition among children in a remote rural area of Bangladesh J Health Popul Nutr 2000 18 123 130 11262764

[B48] Darnton-Hill I Webb P Harvey PWJ Hunt JM Dalmiya N Chopra M Ball MJ Bloem MW de Benoist B Micronutrient deficiencies and gender: social and economic costs Am J Clin Nutr 2005 81 1198S 1205S 15883452 10.1093/ajcn/81.5.1198

[B49] Pell JP Pell ACH Dunn F Oldroyd K MacIntyre P O'Rourke B Borland W Haw S Cobbe S Newby DE Fischbacher C McConnachie A Pringle S Murdoch D Smoke-free legislation and hospitalizations for acute coronary syndrome N Engl J Med 2008 359 482 491 10.1056/NEJMsa0706740 18669427

[B50] Sims M Maxwell R Bauld L Gilmore A Short term impact of smoke-free legislation in England: retrospective analysis of hospital admissions for myocardial infarction BMJ 2010 340 c2161 10.1136/bmj.c2161 20530563 PMC2882555

[B51] Ferrante D Linetzky B Virgolini M Schoj V Apelberg B Reduction in hospital admissions for acute coronary syndrome after the successful implementation of 100% smoke-free legislation in Argentina: a comparison with partial smoking restrictions Tob Control 2012 21 402 406 10.1136/tc.2010.042325 21602536

[B52] Barnoya J Glantz S Cardiovascular effects of secondhand smoke: nearly as large as smoking Circulation 2005 111 2684 2698 10.1161/CIRCULATIONAHA.104.492215 15911719

[B53] Been JV Nurmatov UB Cox B Nawrot TS Schayck CP Sheikh A Effect of smoke-free legislation on perinatal and child health: a systematic review and analysis Lancet 2014 http://www.thelancet.com/journals/lancet/article/PIIS0140-6736(14)60082-9/fulltext 10.1016/S0140-6736(14)60082-9 24680633

[B54] World Health Organization The World Health Report: Health Systems Financing: The Path to Universal Coverage 2010 Geneva: World Health Organization 10.2471/BLT.10.078741 PMC2878164 20539847

[B55] United Nations Economic and Social Council General Comment No. 14. The Right to the Highest Attainable Standard of Health 2000 Geneva: United Nations Economic and Social Council

[B56] Backman G Frisancho A Tarco D Motlagh M Farcasanu D Vladescu C Hunt P Khosla R Jaramillo-Strouss C Fikre BM Rumble C Pevalin D Páez DA Pineda MA Health systems and the right to health: an assessment of 194 countries Lancet 2008 372 2047 2085 10.1016/S0140-6736(08)61781-X 19097280

[B57] World Health Organization Package of Essential Noncommunicable (PEN) Disease Interventions for Primary Health Care in Low Resource Settings 2010 Geneva: World Health Organization

[B58] Lim S Gaziano T Gakidou E Reddy K Farzadfar F Lozano R Rodgers A Prevention of cardiovascular disease in high-risk individuals in low-income and middle-income countries: health effects and costs Lancet 2007 370 2054 2062 10.1016/S0140-6736(07)61699-7 18063025

[B59] Yusuf S Avezum A Kruger A Kutty R Lanas F Lisheng L Wei L Lopez-Jaramillo P Oguz A Rahman O Swidan H Islam S Yusoff K Zatonski W Rosengren A Teo KK Chow CK Rangarajan S Dagenais G Diaz R Gupta R Kelishadi R Iqbal R Use of secondary prevention drugs for cardiovascular disease in the community in high-income, middle-income, and low-income countries (the PURE Study): a prospective epidemiological survey Lancet 2011 378 1231 1243 10.1016/S0140-6736(11)61215-4 21872920

[B60] Niëns L Cameron A Van de Poel E Ewen M Brouwer W Laing R Quantifying the impoverishing effect of purchasing medicines: a cross-country comparison of the affordability of medicines in the developing world PLoS Med 2010 7 e1000333 10.1371/journal.pmed.1000333 20824175 PMC2930876

[B61] Cameron A Ewen M Ross-Degnan D Ball D Laing R Medicine prices, availability, and affordability in 36 developing and middle-income countries: a secondary analysis Lancet 2009 373 240 249 10.1016/S0140-6736(08)61762-6 19042012

[B62] Mendis S Fukino K Cameron A Laing R Filipe A Jr Khatib O Leowski J Ewen M The availability and affordability of selected essential medicines for chronic diseases in six low- and middle-income countries Bull World Health Organ 2007 85 279 288 10.2471/BLT.06.033647 17546309 PMC2636320

[B63] World Health Organization The World Health Report, 2008: Primary Health Care (Now, More Than Ever) 2008 Geneva: World Health Organization

[B64] Samb B Patel K Adshead F McKee M Evans T Alwan A Etienne C Desai N Nishtar S Mendis S Bekedam H Wright A Hsu J Martiniuk A Celletti F Chronic diseases: chronic diseases and development 4 prevention and management of chronic disease: a litmus test for health-systems strengthening in low-income and middle-income countries Lancet 2010 376 1785 1797 10.1016/S0140-6736(10)61353-0 21074253

[B65] World Health Organization Everybody’s Business: Strengthening Health Systems to Improve Health Outcomes: WHO’s Framework for Action 2007 Geneva [http://www.who.int/healthsystems/strategy/en/]

[B66] Kutzin J Health financing for universal coverage and health system performance: concepts and implications for policy Bull World Health Organ 2013 91 62 611 10.2471/BLT.12.113985 PMC3738310 23940408

[B67] Kinney E Clark B Provisions for health and health care in the constitutions of the countries of the world Cornell Int Law J 2004 37 285 355

[B68] Sentencia T-760/08 Acciones de Tutela Instauradas por Luz Mary Osorio Palacio Contra Colpatria EPS Colombia: Corte Constitucional de Colombia 31 julio 2008 (expediente T-1281247)

[B69] See Azanca Alheli Meza García Peru: Tribunal Constitucional de Peru 20 abril 2004 (expediente 2945-2003-AA/TC)

[B70] Sentencia 196 Cruz del Valle Bermúdez y otros v. MSAS s/amparao Venezuela: Tribunal Supreme de Venezuela 15 mayo 1999 (expediente 15.789)

[B71] Puhl R Heuer C The stigma of obesity: a review and update Obesity 2009 17 941 964 10.1038/oby.2008.636 19165161

[B72] Kalra B Kalra S Kumar A Social stigma and discrimination: a care crisis for young women with diabetes in India Diabetes Voice 2009 54 Special Issue 37 39

[B73] World Health Organization Medium-Term Strategic Plan 2008–2013 [http://apps.who.int/gb/ebwha/pdf_files/MTSP-08-13-PPB-10-11/mtsp-3en.pdf]

[B74] Abegunde D Essential medicines for non-communicable diseases (NCDs). Background paper http://www.who.int/medicines/areas/policy/access_noncommunicable/EssentialMedicinesforNCDs.pdf

[B75] Agreement on Trade-Related Aspects of Intellectual Property Rights: Marrakesh Agreement Establishing the World Trade Organization Annex 1C, The legal texts: the results of the Uruguay round of multilateral trade negotiations 321 (1999), 1869 UNTS 299 [http://www.wto.org/english/docs_e/legal_e/legal_e.htm#TRIPs]

[B76] Musungu S Oh C The use of flexibilities in TRIPS by developing countries: can they promote access to medicines? Geneva: World Health Organization; 2005 [http://www.who.int/intellectualproperty/studies/TRIPSFLEXI.pdf]

[B77] Abbott F van Puymbroeck R Compulsory licensing for public health: a guide and model documents for implementation of the DOHA Declaration Paragraph 6 Decision. Washington DC: The World Bank; 2005 [http://documents.worldbank.org/curated/en/2005/07/6258659/compulsory-licensing-public-health-guide-model-documents-implementation-doha-declaration-paragraph-6-decision]

[B78] Van Puymbroeck R Basic survival needs and access to medicines – coming to grips with TRIPS: conversion + calculation J Law Med Ethics 2010 38 520 549 10.1111/j.1748-720X.2010.00510.x 20880239

[B79] Nicol D Owoeye O Using TRIPS flexibilities to facilitate access to medicines Bull World Health Organ 2013 91 533 539 10.2471/BLT.12.115865 23825881 PMC3699798

[B80] United Nations General Assembly Political Declaration of the High-level Meeting of the General Assembly on the Prevention and Control of Non-communicable Diseases. A/66/L.1 (New York, 16 September 2011)

[B81] Bollyky T Access to drugs for treatment of noncommunicable diseases PLoS Med 2013 10 7 e10011485 10.1371/journal.pmed.1001485 PMC3720246 23935456

[B82] Gabble R Kohler JC ‘To patent or not to patent?’ the case of Novartis’ cancer drug Glivec in India Glob Health 2014 10 3 [http://www.globalizationandhealth.com/content/10/1/3] 10.1186/1744-8603-10-3 PMC3884017 24393270

[B83] Glassman A Duran D Sumner A Global health and the new bottom billion: what do shifts in global poverty and disease burden mean for donor agencies? Global Policy 2013 4 1

[B84] World Health Organization Ensuring Balance in National Policies on Controlled Substances: Guidance for Availability and Accessibility of Controlled Medicines 2011 Geneva: World Health Organization

[B85] Lohman D Schleifer R Amon J Access to pain treatment as a human right BMC Med 2010 8 8 10.1186/1741-7015-8-8 20089155 PMC2823656

[B86] United Nations Pain initiative at the United Nations [http://www.pain-initiative-un.org/pain-initiative4/]

[B87] International Association for the Study of Pain Declaration of Montréal, adopted 3 September 2010 [http://www.iasp-pain.org/Advocacy/Content.aspx?ItemNumber=1821]

[B88] Liberman J Implications of international law for the treatment of cancer: the single convention on narcotic drugs and the TRIPS agreement Public Health 2011 125 840 846 10.1016/j.puhe.2011.09.032 22054908

[B89] World Health Organization WHO Report on the Global Tobacco Epidemic 2013 Geneva

[B90] Garrett L So, What Was Accomplished? The UN and NCDs [http://lauriegarrett.com/blog/2011/9/22/so-what-was-accomplished-the-un-and-ncds]

[B91] Rabkin M El-Sadr W Why reinvent the wheel? Leveraging the lessons of HIV scale-up to confront non-communicable diseases Glob Public Health 2011 6 247 256 10.1080/17441692.2011.552068 21390970

[B92] Stuckler D Nestle M Big food, food systems, and global health PLoS Med 2012 9 6 e1001242 10.1371/journal.pmed.1001242 22723746 PMC3378592

[B93] Moodie R Swinburn B Richardson J Somaini B Childhood obesity – a sign of commercial success, but a market failure Int J Pediatr Obes 2006 1 133 138 10.1080/17477160600845044 17899630

[B94] Stuckler D McKee M Ebrahim S Basu S Manufacturing epidemics: the role of global producers in increased consumption of unhealthy commodities including processed foods, alcohol, and tobacco PLoS Med 2012 9 6 e1001245 22745605 10.1371/journal.pmed.1001235 PMC3383750

[B95] Labonté R Mohindra K Lenchucha R Framing international trade and chronic disease Global Health 2011 7 21 10.1186/1744-8603-7-21 21726434 PMC3158109

[B96] Hawkes C Chopra M Friel S Labonté R, Schrecker T, Packer C, Runnels V Globalization, Trade and the Nutrition Transition Globalization and Health: Pathways, Evidence and Policy 2009 New York: Routledge 235 262

[B97] Hastings G Why corporate power is a public health priority BMJ 2012 34 e5124 22915664 10.1136/bmj.e5124

[B98] Food Industry Asia The contribution of food & beverage companies to health and nutrition in Asia. Summary of initiatives – 2012 [https://foodindustry.asia/documentdownload.axd?documentresourceid=379]

[B99] Kusnandar & Co It’s not necessary for Indonesia to ratify the FCTC [blogpost]. 9 January 2014 [http://kusnandarlaw.blogspot.com.au/2014/01/its-not-necessary-yet-for-indonesia-to.html]

[B100] Friel S Gleeson D Thow A-M Labonte R Stuckler D Kay A Snowdon W A new generation of trade policy: potential risks to diet-related health from the trans pacific partnership agreement Global Health 2013 9 46 10.1186/1744-8603-9-46 24131595 PMC4016302

[B101] Thow AM McGrady B Protecting policy space for public health nutrition in an era of international investment agreements Bull World Health Organ 2014 92 139 145 10.2471/BLT.13.120543 24623907 PMC3949531

[B102] Philip Morris Brand Sàrl (Switzerland), Philip Morris Products S.A. (Switzerland) and AbalHermanos S.A. (Uruguay) v. Oriental Republic of Uruguay (ICSID Case No. ARB/10/7) International Centre for Settlement of Investor Disputes [https://icsid.worldbank.org/ICSID/FrontServlet?requestType=GenCaseDtlsRH&actionVal=ListPending]

[B103] Voon T Mitchell A Liberman J Ayres G Public Health and Plain Packaging of Cigarettes 2012 Cheltenham: Edward Elgar Publishing

[B104] Daube M Stafford J Bond L No need for Nanny Tob Control 2008 17 426 427 10.1136/tc.2008.027763 19029365

[B105] Jochelson K Nanny or steward? The role of government in public health Public Health 2006 120 1149 1155 10.1016/j.puhe.2006.10.009 17067645

[B106] Brownell K Kersh R Ludwig D Post R Puhl R Schwarts M Willett W Personal responsibility and obesity: a constructive approach to a controversial issue Health Aff 2010 29 379 387 10.1377/hlthaff.2009.0739 20194976

[B107] Hitchens C I fought the law Vanity Fair 2004 74 79

[B108] Hall M The scope and limits of public health Perspect Biol Med 2003 46 3 S199 S209 10.1353/pbm.2003.0054 14563083

[B109] Calman K Beyond the ‘Nanny State’: stewardship and public health Public Health 2009 123 e6 e10 19135693 10.1016/j.puhe.2008.10.025 PMC7118790

[B110] Wikler D Personal and social responsibility for health Ethics Int Aff 2002 16 47 55 10.1111/j.1747-7093.2002.tb00396.x 15709278

[B111] Magnusson R Bloomberg, Hitchins, and the Libertarian Critique Hastings Cent Rep 2014 44 1 3 4 24408587 10.1002/hast.242

[B112] Glantz S Gonzalez M Effective tobacco control is key to rapid progress in reduction of non-communicable diseases Lancet 2012 379 1269 1271 10.1016/S0140-6736(11)60615-6 21963004 PMC3260384

[B113] Zhu C Young-soo S Beaglehole R Tobacco control in China: small steps towards a giant leap Lancet 2012 379 779 780 10.1016/S0140-6736(11)61933-8 22386013

[B114] World Health Organization Regional Office for South-East Asia. Noncommunicable Diseases in the South-East Asia Region, 2011 – Situation and Response 2011 Geneva: World Health Organization

[B115] Djono A Indonesia ‘blowing smoke’ over tobacco control *Jakarta Globe.* 8 November 2013. [http://www.thejakartaglobe.com/news/indonesia-blowing-smoke-over-tobacco-control/]

[B116] World Health Organization MPOWER: a policy package to reverse the tobacco epidemic [http://www.who.int/tobacco/mpower/en/]

[B117] Méndez D Alshanqeety O Warner K The potential impact of smoking control policies on future global smoking trends Tob Control 2012 22 46 51 22535364 10.1136/tobaccocontrol-2011-050147

[B118] Beaglehole R Bonita R Horton R Ezzati M Bhala N Amuyunzu-Nyamongo M Mwatsama M Reddy KS Measuring progress on NCDs: one goal and five targets Lancet 2012 380 1283 1285 10.1016/S0140-6736(12)61692-4 23063272

[B119] Capewell S O’Flaherty M Rapid mortality falls after risk-factor changes in populations Lancet 2011 378 752 753 10.1016/S0140-6736(10)62302-1 21414659

[B120] Institute of Medicine Country-Level Decision-Making for Control of Chronic Diseases. Workshop Summary 2012 Washington DC: The National Academies Press 24830055

[B121] Kelly B Pérez Koehlmoos T Nugent R Exploring country-level decision making for the control of chronic diseases Global Heart 2012 7 1 7 12 10.1016/j.gheart.2012.02.004 25691164

[B122] Hawkes C Jewell J Allen K A food policy package for healthy diets and the prevention of obesity and diet-related non-communicable diseases: the NOURISHING framework Obes Rev 2013 14 Suppl 2 159 168 24103073 10.1111/obr.12098

[B123] Gostin L Bloomberg’s Health Legacy: Urban Innovator or Meddling Nanny Hastings Cent Rep 2013 43 5 19 25 24092587 10.1002/hast.208

[B124] Lorillard Tobacco Co. v Reilly 121 S. Ct. 2404 (2001) (U.S. Supreme Court); 44 Liquormart, Inv. v Rhode Island 517 U.S. 484 (1996) (U.S. Supreme Court)

[B125] R.J. Reynolds Tobacco Co. V Food & Drug Administration U.S. Court of Appeals for the District of Columbia 24 August 2012. No. 11–5332

[B126] Gostin L Public Health Law: Power, Duty, Restraint 2008 Berkeley: University of California Press

[B127] Berman M Commercial speech law and tobacco marketing: a comparative discussion of the United States and Canada Am J Law Med 2013 39 218 236 23815029 10.1177/009885881303900202

[B128] Cabrera O Madrazo A Human rights as a tool for tobacco control in Latin America Salud Publica Mex 2010 52 suppl 2 S288 S297 21243201 10.1590/s0036-36342010000800026

[B129] Murli S. Deora v. Union of India, 8 SSC 765 Supreme Court of India 2001

[B130] Cigarettes and Other Tobacco Products Act 2003 *Cigarettes and Other Tobacco Products Act* 2003 (India) [http://www.tobaccocontrollaws.org/legislation/country/India]

[B131] Campaign for Tobacco-Free Kids International Legal Consortium. Litigation Project [http://www.tobaccocontrollaws.org/litigation]

[B132] Australian Associated Press Mild, light cigarette descriptions axed The Sydney Morning Herald 7 November 2005

[B133] Rani M Nusrat S Hawken L A qualitative study of governance of evolving response to non-communicable diseases in low- and middle-income countries: current status, risks and options BMC Public Health 2012 12 877 10.1186/1471-2458-12-877 23067232 PMC3487912

[B134] Borland R Winstanley M Reading D Legislation to institutionalize resources for tobacco control: the 1987 Victorian Tobacco Act Addiction 2009 104 1623 1629 10.1111/j.1360-0443.2009.02701.x 21265905

[B135] Brownell K Farley T Willett W Popkin B Chaloupka F Thompson J Ludwig D The public health and economic benefits of taxing sugar-sweetened beverages N Engl J Med 2009 361 1599 1605 10.1056/NEJMhpr0905723 19759377 PMC3140416

[B136] Claro R Levy R Popkin B Monteiro C Sugar-sweetened beverage taxes in Brazil Am J Pub Health 2012 102 178 183 10.2105/AJPH.2011.300313 22095333 PMC3490548

[B137] Briggs A Mytton O Kehlbacher A Tiffin R Rayner M Scarborough P Overall and income specific effect on prevalence of overweight and obesity of 20% sugar sweetened drink tax in the UK: econometric and comparative risk assessment modelling study BMJ 2013 347 f6189 10.1136/bmj.f6189 24179043 PMC3814405

[B138] ACUERDO por el que se crea el Consejo Nacionalpara la Prevención y Control de las Enfermeda des Crónicas No Transmisibles. 11 February 2010 [http://dof.gob.mx/nota_detalle.php?codigo=5131456&fecha=11/02/2010]

[B139] Patient Protection and Affordable care act (P.L. 111–148), Title IV (§4001); Executive Order 13544. Establishing the National Prevention, Health Promotion, and Public Health Council. 10 June 2010 [http://www.whitehouse.gov/the-press-office/executive-order-establishing-national-prevention-health-promotion-and-public-health]

[B140] National Prevention Council [http://www.surgeongeneral.gov/initiatives/prevention/about/index.html]

[B141] National Prevention Council National Prevention Council Action Plan: Implementing the National Prevention Strategy [http://www.surgeongeneral.gov/initiatives/prevention/about/actionplan.html]

[B142] UNAIDS UN support to country level responses to HIV/AIDS: an in-depth assessment of UN Theme Groups on HIV/AIDS [http://www.undg.org/content/programming_reference_guide_%28undaf%29/thematic_policies_and_guidelines]

[B143] UNAIDS Resource Guide for UN Theme Groups on HIV/AIDS 2005 Geneva: UNAIDS

[B144] Editorial Alcohol misuse needs a global response Lancet 2007 373 433 10.1016/S0140-6736(09)60146-X 19200899

[B145] Editorial Urgently needed: a framework convention on obesity control Lancet 2011 378 741 10.1016/S0140-6736(11)61356-1 21872731

[B146] Gostin LO Friedman EA Buse K Waris A Mulumba M Joel M Dare L Dhai A Sridhar D Towards a framework convention on global health Bull World Health Organ 2013 91 790 793 10.2471/BLT.12.114447 24115803 PMC3791648

[B147] United Nations General Assembly The future we want. A/RES/66/288 [http://www.uncsd2012.org/thefuturewewant.html]

[B148] United Nations A new global partnership: eradicate poverty and transform economies through sustainable development. The report of the high-level panel of eminent persons on the post-2015 development agenda New York; 2013

[B149] World Health Assembly: Follow-up to the Political Declaration of the High-level Meeting of the General Assembly on the Prevention and Control of Noncommunicable Diseases (WHA66.10, 27 May 2013) (annexing the Global Action Plan for the Prevention and Control of Noncommunicable Diseases 2013–2020)

[B150] United Nations Economic and Social Council United Nations Interagency Task Force on the Prevention and Control of Non-communicable Diseases, E/2013/L.23 2013 Geneva: United Nations

[B151] World Health Organization Global Action Plan for the Prevention and Control of Noncommunicable Diseases 2013–2020, WHA66.10 2013 Geneva: World Health Organization

[B152] World Health Organization UN Interagency Task Force on the Prevention and Control of NCDs [website] [http://www.who.int/nmh/events/ncd_task_force/en/]

[B153] World Health Organization Draft terms of reference for the United Nations Interagency Task Force on the Prevention and Control of Noncommunicable Diseases WHO Discussion Paper (2 September 2013)

[B154] World Health Organization Terms of reference for the United Nations Interagency Task Force on the Prevention and Control of Noncommunicable Diseases, including a division of tasks and responsibilities for UN organizations and other intergovernmental organizations WHO Discussion Paper (4 October 2013)

[B155] World Health Organization Draft terms of reference for a global coordination mechanism for the prevention and control of noncommunicable diseases WHO Discussion Paper (23 July 2013)

[B156] World Health Organization Global coordination mechanism for NCDs [website] [http://www.who.int/nmh/events/ncd_coordination_mechanism/en/]

[B157] The global fund to fight AIDS, tuberculosis and malaria [http://www.theglobalfund.org/en/]

[B158] Institute for Health Metrics and Evaluation Financing Global Health 2012: The end of the Golden age? 2012 Seattle: Institute for Health Metrics and Evaluation

[B159] United Nations General Assembly Note by the Secretary-General transmitting the report of the Director-General of the World Health Organization on options for strengthening and facilitating multisectoral action for the prevention and control of non-communicable diseases through effective partnership A/67/373 (17 September 2012)

[B160] Magnusson R Rethinking global health challenges: towards a ‘global compact’ for reducing the burden of chronic disease Public Health 2009 123 265 274 10.1016/j.puhe.2008.12.023 19278695

[B161] Magnusson R Non-communicable diseases and global health governance: enhancing global processes to improve health development Glob Health 2007 3 2 [http://www.globalizationandhealth.com/content/3/1/2] 10.1186/1744-8603-3-2 PMC1894788 17519005

[B162] World Health Organization Comprehensive Global Monitoring Framework, Including 25 Indicators, and a set of Nine Voluntary Global Targets for the Prevention and Control of Noncommunicable Diseases 2013 Geneva: World Health Organization

[B163] World Health Organization Set of action plan indicators for the WHO Global NCD Action Plan 2013–2020 [http://www.who.int/nmh/events/action_plan_indicators/en/index.html]

[B164] World Health Organization Country profile of capacity and responses to noncommunicable diseases (NCDs), 2013 [http://www.who.int/chp/ncd_capacity/CCS_2013_Questionnaire.pdf]

[B165] Swinburn B Sacks G Vandevijvere S Kumanyika S Lobstein T Neal B Barquera S Friel S Hawkes C Kelly B L'Abbe M Lee A Ma J Macmullan J Mohan S Monteiro C Rayner M Sanders D Snowdon W Walker C INFORMAS: (International network for food and obesity/non-communicable diseases research, monitoring and action support): overview and key principles Obes Rev 2013 14 Suppl 1 1 12 10.1111/obr.12087 24074206

[B166] INFORMAS Benchmarking food environments [website] [http://www.fmhs.auckland.ac.nz/soph/globalhealth/informas/]

[B167] UNAIDS Global AIDS response progress reporting [http://www.unaids.org/en/dataanalysis/knowyourresponse/globalaidsprogressreporting/]

[B168] UNAIDS Global AIDS Response Progress Reporting 2013: Construction of Core Indicators for Monitoring the 2011 UN Political Declaration on HIV/AIDS 2013 Geneva: UNAIDS

[B169] United Nations General Assembly General Assembly Special Session on HIV/AIDS. 25–27 June 2001 [http://www.un.org/ga/aids/coverage/]

[B170] United Nations General Assembly Political Declaration on HIV and AIDS: Intensifying our Efforts to Eliminate HIV and AIDS 2011 New York: United Nations

[B171] UNAIDS Global AIDS Response Progress Reporting 2012: Guidelines: Construction of Core Indicators for Monitoring the 2011 UN Political Declaration on HIV/AIDS 2011 Geneva: UNAIDS

[B172] Taylor AL Alfven T Hougendobler D Tanaka S Buse K Leveraging non-binding instruments for global health governance: reflections from the Global AIDS Reporting Mechanism for WHO Reform Public Health 2013 128 151 160 24393497 10.1016/j.puhe.2013.08.022

[B173] World Health Organization Discussion paper. Development of an updated Action Plan for the Global Strategy for the Prevention and Control of Noncommunicable Diseases Covering the Period 2013 to 2020. 26 July 2012 [http://www.who.int/nmh/events/2012/ncd_action_plan/en/index.html]

[B174] Magnusson R Patterson D Role of law in global response to non-communicable diseases Lancet 2011 378 859 860 10.1016/S0140-6736(11)60975-6 21890035

[B175] Attaran A Pang T Whitworth J Oxman A McKee M Health by law: the missed opportunity to use laws for public health Bull World Health Organ 2012 379 283 285 10.1016/S0140-6736(11)60069-X 21982516

[B176] Moodie R Stuckler D Monteiro C Sheron N Neal B Thamarangsi T Lincoln P Casswell S on behalf of the Lancet NCD Action Group Profits and pandemics: prevention of harmful effects of tobacco, alcohol, and ultra-processed food and drink industries Lancet 2013 381 670 679 10.1016/S0140-6736(12)62089-3 23410611

[B177] Bryden A Petticrew M Mays N Eastmure E Knai C Voluntary agreements between government and business – a scoping review of the literature with specific reference to the Public Health Responsibility Deal Health Policy 2013 110 186 197 10.1016/j.healthpol.2013.02.009 23506799

[B178] Marks J What’s the big deal? The ethics of public-private partnerships related to health [http://papers.ssrn.com/sol3/papers.cfm?abstract_id=2268079] 10.1353/ken.2014.0022 25423851

[B179] Sharma L Teret S Brownell K The food industry and self-regulation: standards to promote success and to avoid public health failures Am J Public Health 2010 100 240 246 10.2105/AJPH.2009.160960 20019306 PMC2804645

[B180] Hashem K Haigh C Powell C The irresponsibility deal? [http://www.sustainweb.org/publications/?id=188]

[B181] Panjwani C Caraher M The public health responsibility deal: brokering a deal for public health, but on whose terms? Health Policy 2014 114 163 173 10.1016/j.healthpol.2013.11.002 24309298

[B182] Framework Convention Alliance [http://www.fctc.org]

